# Synthesis and Evaluation of New Halogenated GR24 Analogs as Germination Promotors for *Orobanche cumana*

**DOI:** 10.3389/fpls.2021.725949

**Published:** 2021-09-17

**Authors:** Yuchao Chen, Yi Kuang, Liyang Shi, Xing Wang, Haoyu Fu, Shengxiang Yang, Diego A. Sampietro, Luqi Huang, Yuan Yuan

**Affiliations:** ^1^School of Pharmacy, Anhui University of Chinese Medicine, Hefei, China; ^2^State Key Laboratory of Dao-di Herbs Breeding Base, National Resources Center for Chinese Materia Medica, China Academy of Chinese Medical Sciences, Beijing, China; ^3^Agricultural Biotechnology Center, Ningxia Academy of Agriculture and Forestry Sciences, Yinchuan, China; ^4^Zhejiang Provincial Collaborative Innovation Center for Bamboo Resources and High-Efficiency Utilization, Zhejiang A&F University, Lin’an, China; ^5^LABIFITO, National University of Tucumán, Tucumán, Argentina

**Keywords:** strigolactones, *Orobanche cumana*, parasitic weeds, GR24 analogs, suicidal germination

## Abstract

*Orobanche* and *Striga* are parasitic weeds extremely well adapted to the life cycle of their host plants. They cannot be eliminated by conventional weed control methods. Suicidal germination induced by strigolactones (SLs) analogs is an option to control these weeds. Here, we reported two new halogenated (+)-GR24 analogs, named 7-bromo-GR24 (7BrGR24) and 7-fluoro-GR24 (7FGR24), which were synthesized using commercially available materials following simple steps. Both compounds strongly promoted seed germination of *Orobanche cumana*. Their EC_50_ values of 2.3±0.28×10^−8^M (7BrGR24) and 0.97±0.29×10^−8^M (7FGR24) were 3- and 5-fold lower, respectively, than those of (+)-GR24 and *rac*-GR24 (EC_50_=5.1±1.32–5.3±1.44×10^−8^; *p*<0.05). The 7FGR24 was the strongest seed germination promoter tested, with a stimulation percentage of 62.0±9.1% at 1.0×10^−8^M and 90.9±3.8% at 1.0×10^−6^M. It showed higher binding affinity (IC_50_=0.189±0.012μM) for the SL receptor ShHTL7 than (+)-GR24 (IC_50_=0.248±0.032μM), *rac*-GR24 (IC_50_=0.319±0.032μM), and 7BrGR24 (IC_50_=0.521±0.087μM). Molecular docking experiments indicated that the binding affinity of both halogenated analogs to the strigolactone receptor OsD14 was similar to that of (+)-GR24. Our results indicate that 7FGR24 is a promising agent for the control of parasitic weeds.

## Introduction

The parasitic weeds *Orobanche* spp. (broomrapes) and *Striga* spp. (witchweeds) can feed through haustoria invading the roots of host plants (Musselman, 1980). They parasitize major crops, including maize (*Zea mays*), sorghum (*Sorghum bicolor*), rice (*Oryza sativa*), tomato (*Lycopersicon esculentum*), tobacco (*Nicotiana tabacum*), and sunflower (*Helianthus annuus*). *Orobanche* and *Striga* species infest more than 60 million hectares of farmland worldwide, resulting in the loss of billions of dollars each year (Chesterfield et al., 2020). For instance, approximately 1.34 million hectares of rain-fed rice field in Africa are infested with *Striga*, resulting in crop losses of more than USD100 million. These weeds cause economic pressure on millions of smallholder farmers (Parker, 2012; Rodenburg et al., 2016). These parasitic weeds are expanding their geographical range. *Orobanche cumana* was first reported on sunflowers in central Russia at the end of the 19th century. It spread over east Europe in a few decades along with the successful expansion of sunflower harvests. It is currently found in most of the main sunflower-producing countries in Eurasia, from Spain to China and is regarded as the most important biotic constraint for sunflower production (Rubiales, 2020).

*Orobanche* and *Striga* weeds are not effectively controlled by conventional methods, such as breeding resistant varieties, rotation, and herbicides (Hearne, 2009). Their plants produce tens of thousands of tiny seeds that remain viable and dormant for over 10years and lead to the formation of extensive seed stocks in the soil (Musselman, 1980). The seeds only germinate in response to specific germination signals, known as strigolactones (SLs), which are released in the rhizosphere by the host plants. Strigol was the first SL identified (Cook et al., 1967). Since then, more than 20 SLs have been isolated from host crop plants, including sorghum, maize, rice, and tobacco (Hauck et al., 1992; Siame et al., 1993; Xie et al., 2013). Molecules of these natural SLs are composed of a tricyclic lactone ring (ABC-ring) and a butenolide ring (D-ring) that are connected by an enol-ether linkage, where the bioactiphore for germination resides in the CD part (Zwanenburg et al., 2009; Zwanenburg and Blanco-Ania, 2018). *Orobanche* and *Striga* weeds need their plant hosts to survive. Hence, the application of SLs to soils infested with parasitic weeds is a promising alternative to stimulate suicidal seed germination before the crop is planted (Zwanenburg et al., 2016). However, natural SLs found in root exudates are available at picogram levels and have an unstable structure (Yoneyama et al., 2013). Therefore, synthetic analogs, such as GR24, GR7, GR5, Nijmegen-1, and T-010, were synthesized. They offer interesting prospects for eliminating parasitic weeds through suicidal germination (Zwanenburg and Blanco-Ania, 2018). However, most of the synthetic SL analogs promote less seed germination than natural SLs. Thus, modification of synthetic SL analogs for commercial application toward controlling parasitic weeds remains highly desirable. Here, we reported the synthesis of new SLs analogs and their effect on seed germination of parasitic weeds.

## Materials and Methods

### General Experimental Procedure

All reactions requiring anhydrous or inert conditions were carried out under a positive atmosphere of argon in oven-dried glassware. Solutions or liquids were introduced into round-bottomed flasks using oven-dried syringes through rubber septa. All reactions were stirred magnetically using Teflon-coated stirring bars. If needed, reactions were warmed using an electrically heated silicon oil bath. Organic solutions obtained after aqueous workup were dried over MgSO_4_. The removal of solvents was accomplished using a rotary evaporator at water aspirator pressure. GR24 stands for *rac*-GR24, which was purchased from Shanghai Yuanye Biotechnology (Shanghai, China). Chemicals for the syntheses were purchased from Sigma-Aldrich (Shanghai, China).

NMR spectra were recorded on Bruker ADVANCE III (400MHz) spectrometers (Karlsruhe, Germany) for ^1^H NMR and ^13^C NMR. CD_3_OD and CDCl_3_ were used as solvents for the NMR analysis, with tetramethylsilane as the internal standard. Chemical shifts were reported upfield to TMS (0.00ppm) for ^1^H NMR and relative to CDCl_3_ (77.3ppm) for ^13^C NMR. Optical rotation was determined using a Perkin Elmer 343 polarimeter. HPLC analysis was conducted on an Agilent 1260 series instrument (California, America). Column chromatography was performed using silica gel Merck 60 (230–400 mesh). All new products were further characterized by HRMS. A positive ion mass spectrum of the sample was acquired on a Thermo LTQ-FT mass spectrometer (MA, United States) with an electrospray ionization source.

### Synthesis of (+)-GR24

**Figure 1:**
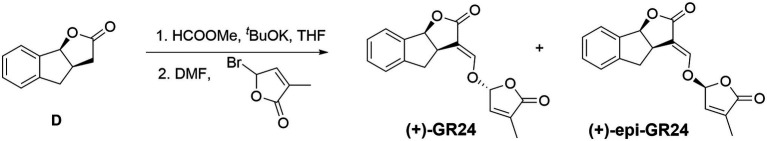
Synthesis of (+)-GR24.

Small portions of potassium tert-butoxide (0.85g, 7.56mmol) were added to a solution of compound D (1.1g, 6.3mmol) and methyl formate (0.82ml, 9.45mmol) in anhydrous THF (15ml) at 0°C under nitrogen (Figure 1). The reaction mixture was stirred at 25°C until completion. THF was removed *in vacuo*. The resulting solid was solubilized in 20ml anhydrous DMF under N_2_. Bromobutenolide (1.67g, 9.45mmol) was added to this solution and the reaction mixture was stirred overnight. The reaction was quenched with saturated aqueous ammonium chloride (20ml). The reaction mixture was diluted with ethyl acetate (50ml) and washed with water (3×30ml). The organic extract was then washed with brine, dried with Na_2_SO_4_, and the solvent removed under vacuum. The residue was finally purified by silica gel column chromatography (eluent: petroleum ether/ethyl acetate=3:1, *v*/*v*) to give the(+)-GR24.

### Synthesis of (−)-epi-GR24

**Figure 2:**
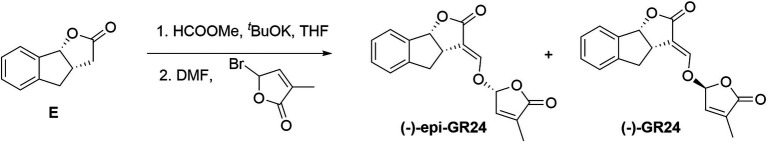
Synthesis of (−)-epi-GR24.

The synthetic protocol described for (+)-GR24 was carried out starting with compound E (1.1g, 6.3mmol; Figure 2) to yield a residue. The residue was finally purified by silica gel column chromatography (eluent: petroleum ether/ethyl acetate=3:1, *v*/*v*) to give the (−)-epi-GR24.

### Synthesis of 7-bromo-GR24 and 7-fluoro-GR24

**Figure 3:**
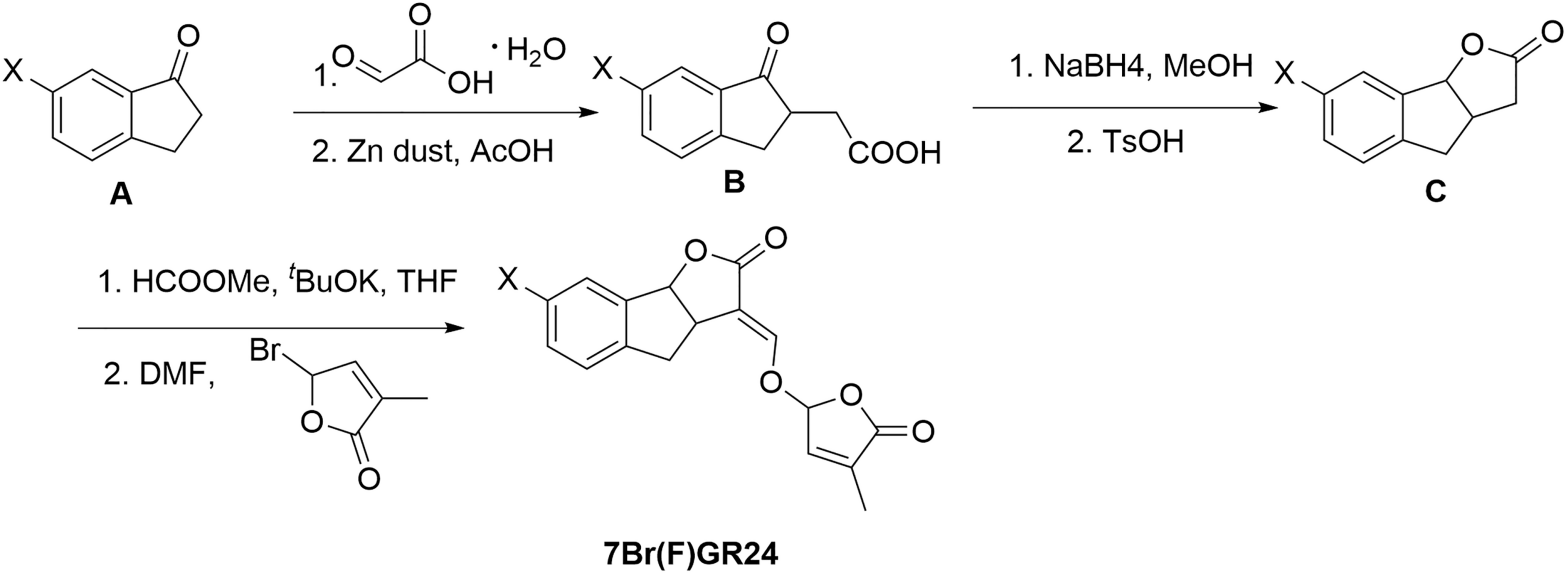
Synthesis of 7-bromo-GR24 (7BrGR24) and 7-fluoro-GR24 (7FGR24), respectively.

Compound A (10mmol of a ketone) and 15mmol of glyoxylic acid were added to a round bottomed flask (Figure 3). Then, the mixture was stirred at 95°C for 3h. The reaction mixture was dissolved in acetic acid (15ml) and water (5ml). Zinc dust (15ml) was added to the solution for 1h and the mixture was stirred for an additional 3h. The mixture was diluted with ethyl acetate and then filtered through celite. The filtrate was extracted with ethyl acetate, washed with brine, dried over Na_2_SO_4_, and concentrated under vacuum. The residue was purified by chromatography on silica gel using hexane:ethyl acetate (2:1, *v*/*v*) and 0.5% acetic acid as an eluent, resulting in 70% yield of compound B.

Compound B (5mmol) was dissolved in 15ml of anhydrous MeOH. Then, 15mmol of NaBH_4_ were added in small portions at 0°C under nitrogen. The reaction mixture was stirred at 25°C until the completion of the reaction. We carefully added 20ml of distilled water to the mixture. The solution was extracted three times with ethyl acetate and the combined organic phase was dried over Na_2_SO_4_, filtered, and concentrated under vacuum. The resulting solid was solubilized in 20ml anhydrous MeOH. TsOH (0.1mmol) was added to this solution and the reaction mixture was stirred at 75°C for 6h. MeOH was removed *in vacuo*. Then, 20ml of distilled water was added. The solution was extracted with ethyl acetate three times and the combined organic phase was dried over Na_2_SO_4_, filtered, and concentrated under vacuum. The residue was purified by chromatography on silica gel using hexane:ethyl acetate (3:1, *v*/*v*) and 0.5% acetic acid as eluents. It yielded 95% of compound C.

The protocol described for (+)-GR24 was performed starting with compound C (1.1g, 6.3mmol) to finally obtain a residue that was subjected to column chromatography, generating pure 7-bromo-GR24 (7BrGR24) and 7-fluoro-GR24 (7FGR24).

### Germination Assays

Seeds of *O. cumana* were kindly provided by professor Yongqing Ma (North-west Agriculture & Forest University, Yangling, China). The assay was carried out in petri dishes according to a method previously reported by Kang et al. (2020). Prior to use, the seeds were sterilized for 8min in 1% sodium hypochlorite, soaked in 75% ethanol for another 1min, rinsed five times with sterile distilled water, and finally left to air dry on a clean bench. A sterile filter paper disk of 6mm in diameter was placed in each petri dish and wetted with 200μl of sterile distilled water. Then, aqueous solutions of the tested compounds (100μl per filter paper) were added. The sterile seeds were distributed on the paper disks at a density of approximately 65 seeds per dish. Finally, the sealed petri dishes were stored in the dark and incubated at 25°C for 14days. After the incubation, the percentage of germination were calculated with the assistance of a stereomicroscope. Compounds 7BrGR24 and 7FGR24 were assayed at concentrations of 1.0×10^−6^, 1.0×10^−7^, 2.0×10^−8^, 1.0×10^−8^, and 1.0×10^−9^M. Three petri dishes were used for each concentration, and assays were carried out three times. The compounds (+)-GR24, (-)-epi-GR24, and *rac*-GR24 were used as the positive control, and the filter paper disk added with 100μl of sterile distilled water was used as the negative control. The EC_50_ values of the tested compounds were calculated with probit tests using SPSS 21.0 software.

### Yoshimulactone Green Assay

The assay was carried out according to a method previously reported by Tsuchiya et al. (2015). The stock solutions of yoshimulactone green (YLG), *rac*-GR24, (+)-GR24, (-)-epi-GR24 and the new SL analogs dissolved in DMSO (1ml, 1mM). Then, stock solutions were diluted with sterile distilled water to final concentrations of 50μM (YLG), and 20, 5, 2.5, 1, 0.5, and 0.1μM [*rac*-GR24, (+)-GR24, (-)-epi-GR24, and the new SL analogs]. Protein coding sequences for ShHTL7 were inserted into KpnI and HindIII sites of the pET32a(+) vector (Invitrogen, CA, United States) and transformed into the *Escherichia coli* strain BL21(DE3; TransGen Biotech, Beijing, China). Briefly, a single colony on the plate was inoculated into a 25ml sterilized LB medium containing 0.28mM ampicillin for 12h. Then, the cultures (1ml) were inoculated into 200ml of the same medium and the cells were grown at 37°C, shaken at 220rpm until an OD (600nm) of 0.6 is reached. We added 0.5mM isopropyl β-D-1-thiogalactopyranoside (IPTG) into the culture to induce protein expression at 16°C for 16h. The culture was centrifuged at 7,000rpm at 4°C for 5min to harvest bacteria. The pellet was suspended in 10ml PBS buffer (6.7mM PO_4_, pH 7.0) containing 1mM PMSF. The cells were lysed using sonication with 5s intervals and centrifuged at 12,000rpm at 4°C for 10min. The supernatant was filtered with a 0.45μm filter and the filtrate was added into a Ni-NTA column (TransGen Biotech, Beijing, China), which had been equilibrated with a PBS buffer. After the Ni-NTA column was washed three times with the PBS buffer, the column was eluted with a 15ml gradient of 20, 50, 100, and 300mM imidazole prepared in the PBS buffer. Fractions from 100mM eluant were pooled for the YLG assay.

The volume of each reaction solution (200μl) contained 5μl of YLG (50μM), 10μl of a dilution of an SL analog, 15μl of ShHTL7 protein (1.5mg/ml), and 170μl of PBS buffer. Reactions were carried out for 3h in the dark in a water bath at 26°C. The blank control contained water (10μl) instead of a dilution of the SL analog. Then, the reaction solutions were added to a 96-well black plate (Nest, Wuxi, China) and its fluorescent intensity was measured by SpectraMax i3 (Molecular Devices, CA, United States) at an excitation wavelength of 480nm and emission wavelength of 520nm. Relative fluorescence units (FU) were calculated as (*k*–*k*')/*k*, where *k* and *k*' are the fluorescence intensities of the blank control and a dilution of the SL analog, respectively. FU were used to calculate IC_50_ values with probit tests using SPSS 21.0 software.

### Molecular Docking Experiment

The molecular modeling computational study was performed using Autodock vina 1.1.2 software. The crystal structure of rice DWARF14 (OsD14; PDB: 5DJ5) was used for the docking study. The grid box was set as a 20×20×20Å three cube and its center was set at the position of the original ligand GR24 (*x*=−30.72, *y*=14.69, and *z*=−21.23). The docking results were visualized by PyMol and Maestro12.7 (for 2D interaction).

### Statistical Analysis

Data of seed germination and fluorescence-based competition assays were subjected to the ANOVA and differences among means were evaluated by the least significant difference test (*p*<0.05).

## Results and Discussion

### Synthesis of SL Analogs

The main features of the synthesized compounds were as follow:

(+)-GR24: White solid, 0.65g, 35% yield, [α]_D_20=+449 (c=0.50, CHCl_3_). ^1^H NMR (400MHz, CDCl_3_) *δ* 7.51–7.49 (m, ^2^H), 7.35 (m, ^3^H), 7.00 (s, ^1^H), 6.21 (s, ^1^H), 5.95 (d, *J*=7.9Hz, ^1^H), 3.97–3.92 (m, ^1^H), 3.44 (dd, *J*=16.9, 9.4Hz, ^1^H), 3.11 (dd, *J*=16.9, 3.2Hz, ^1^H), 2.03 (t, *J*=1.4Hz, ^3^H). ^13^C NMR (100MHz, CDCl_3_) *δ* 171.44, 170.37, 151.27, 142.66, 141.16, 138.82, 135.83, 130.04, 127.49, 126.42, 125.18, 113.14, 100.71, 85.99, 38.85, 37.31, 10.73.

(-)-epi-GR24: White solid, 0.56g, 30% yield, [α]_D_20=−290 (c=0.50, CHCl_3_). ^1^H NMR (400MHz, CDCl_3_) *δ* 7.50–7.49 (m, ^1^H), 7.35–7.23 (m, ^1^H), 6.99 (s, ^1^H), 6.21 (s, ^1^H), 5.96 (d, *J*=8.0Hz, ^1^H), 3.97–3.92 (m, ^1^H), 3.42 (dd, *J*=16.9, 9.3Hz, ^1^H), 3.10 (dd, *J*=16.9, 3.1Hz, ^1^H), 2.03 (t, *J*=1.4Hz, ^3^H). ^13^C NMR (100MHz, CDCl_3_) *δ* 171.42, 170.40, 151.29, 142.69, 141.25, 138.76, 135.76, 130.05, 127.45, 126.34, 125.28, 113.26, 100.79, 86.00, 38.79, 37.42, 29.69, 10.74.

7BrGR24: White solid, mp 195–198°C, 0.83g, 37% yield. ^1^H NMR (400MHz, CDCl_3_) *δ* 7.66 (^1^H, d, *J*=1.2Hz, H-8), 7.51 (^1^H, d, *J*=2.5Hz, H-6'), 7.47 (^1^H, dd, *J*=8.0, 1.7Hz, H-6), 7.13 (^1^H d, *J*=8.0Hz, H-5), 6.97 (^1^H, m, H-3'), 6.21 (^1^H, m, H-2'), 5.93 (^1^H, d, *J*=8.0Hz, H-8b), 3.97 (^1^H, m, H-3a), 3.39 (^1^H, dd, *J*=17.1, 9.3Hz, H-4β), 3.08 (^1^H, dd, *J*=17.0, 3.1Hz, H-4α), 2.07 (^3^H, s, H-7'). ^13^C NMR(100MHz, CDCl_3_) *δ* 170.8 (C-2), 169.9 (C-5'), 151.1 (C-6'), 141.4 (C-8a), 141.1 (C-3'), 140.7 (C-4a), 136.0 (C-8), 134.4 (C-4'), 129.4 (C-5), 126.3 (C-7), 121.0 (C-6), 112.5 (C-3), 100.3 (C-2'), 85.2 (C-8b), 39.2 (C-4), 36.8 (C-3a), 10.8 (C-7'). HR-ESI-MS (*m/z*): calcd. For C_17_H_13_BrNaO_5_ 398.9839; found 398.9835 [M+Na]^+^.

7FGR24: White solid, mp 183–186°C, 0.69g, 35% yield. ^1^H NMR (400MHz, CDCl_3_) *δ* 7.49 (^1^H, d, *J*=2.0Hz, H-8), 7.43 (^1^H, dd, *J*=8.4, 4.4Hz, H-6'), 7.01 (^1^H, m, H-5), 6.97 (^1^H, m, H-6), 6.88 (^1^H, m, H-3'), 6.22 (^1^H, s, H-2'), 5.88 (^1^H, d, *J*=7.9Hz, H-8b), 4.01 (^1^H, m, H-3a), 3.40 (^1^H, dd, *J*=17.2, 9.3Hz, H-4β), 3.07 (^1^H, dd, *J*=17.2, 3.1Hz, H-4α), 2.01 (^3^H, s, H-7'). ^13^C NMR (100MHz, CDCl_3_) *δ* 171.2 (C-2), 170.4 (C-5'), 165.1 (C-7), 151.4 (C-6'), 145.3 (C-8a), 141.2 (C-3'), 135.8 (C-4a), 134.8 (C-4'), 127.9 (C-5), 115.1 (C-8), 112.8 (C-6), 111.9 (C-3), 100.7 (C-2'), 85.0 (C-8b), 39.4 (C-4), 37.3 (C-3a), 10.7 (C-7'). HR-ESI-MS (*m*/*z*): calcd. For C_17_H_13_FNaO_5_ 339.0639; found 339.0642 [M+Na]^+^.

### Seed Germination Assay

Table 1 shows the impact of (+)-GR24, (-)-epi-GR24, *rac*-GR24, 7BrGR24, and 7FGR24 on the seed germination of *O. cumana*. (+)-GR24 and *rac*-GR24 showed a similar stimulatory effect (EC_50_=5.1±1.32–5.3±1.44×10^−8^M), whereas (-)-epi-GR24 had no effect. These results suggested strong stereospecificity in the SL perception of *O. cumana*. (+)-GR24 and (-)-epi-GR24 were diasteroisomers with opposite stereochemistry in the C-ring that is β- and α-oriented, respectively, and had the same 2'-*R* configuration of the D-ring. Indeed, parasitic plant species also vary considerably in their germination responses to different SLs (Wang and Bouwmeester, 2018; Bouwmeester et al., 2021). In general, the 2'-*R* configuration of the D-ring has been confirmed essential for SLs germination activity, and stereochemistry in the C-ring is considered to be closely related to the activity (Thuring et al., 1997a,b; Xie et al., 2010). The use of (+)-GR24 and (-)-epi-GR24 in germination tests could provide profound clues about the general stereochemical adaptation of parasitic weeds for the perception of strigol-like (β-oriented C-ring) and orobanchol-like (α-oriented C-ring) SLs, respectively. These are the two families of natural canonical SLs currently known (Scaffidi et al., 2014; Ueno et al., 2014; Xie, 2016). Seed germination responsiveness reported for either (+)-GR24 or (-)-epi-GR24 exhibited strong variations among the parasitic weed species. For example, *Striga hermonthica* and *Orobanche crenata* germinated when exposed to (+)-GR24 or (-)-epi-GR24 at concentrations between 1×10^−5^ and 1×10^−9^M (Thuring et al., 1997b; Ueno et al., 2011). However, *S. hermonthica* was generally more responsive to (+)-GR24 than *O. crenata* at low concentrations. (-)-epi-GR24 showed lower activity compared to (+)-GR24 on both weed species irrespective of the concentration tested (Thuring et al., 1997b). In contrast, *Orobanche minor* seed showed a higher germination rate when exposed to orobanchol (orobanchol-like SLs) compared with strigol (strigol-like SLs; Xie et al., 2010). Furthermore, *S. hermonthica* could respond to 36 stereoisomers of the naturally occurring SLs including both strigol-like and orobanchol-like SLs, while *Striga gesnerioides* only responded to three orobanchol-like SLs of them (Nomura et al., 2013). These facts comfirmed that some parasitic plant species possessed the strict structural requirements of SLs for induction of germination (Ueno et al., 2011). The differential responsiveness of the parasitic plant species to SLs could be an adaptation to avoid being triggered to germinate by non-host plants (Nomura et al., 2013). Hence, the specific stereochemistry recognition of SLs analogs observed for *O. cumana* in this work was likely due to its host specificity, which was restricted to a short number of plant species (Fernández-Aparicio et al., 2011).

**Table 1:** Values of half-maximal effective concentration (EC_50_) calculated for germination of *Orobanche cumana* seeds exposed to increasing concentrations of (+)-GR24, (−)-epi-GR24, *rac*-GR24, and the halogenated (+)-GR24 analogs.

Compounds	Concentration (M)					EC_50_ (10^−8^M)
	1.0×10^−9^	1.0×10^−8^	2.0×10^−8^	1.0×10^−7^	1.0×10^−6^	
(+)-GR24	7.3±5.9%^a^	21.5±13.4%^c^	41.1±10.1%^b^	66.6±9.1%^c^	81.5±3.9%^b^	5.1±1.32
(−)-epi-GR24	0^b^	0^d^	0^c^	0^d^	0^d^	–
7-bromo-GR24	16.9±4.1%^a^	39.6±5.0%^b^	49.1±6.6%^b^	69.6±3.3%^b^	83.8±2.5%^b^	2.3±0.28
7-fluoro-GR24	11.2±7.8%^a^	62.0±9.1%^a^	63.8±4.9%^a^	84.2±8.6%^a^	90.9±3.8%^a^	0.97±0.29
*rac*-GR24	15.3±8.5%^a^	27.2±4.2%^c^	47.1±5.0%^b^	58.5±10.7%^c^	75.2±5.7%^c^	5.3±1.44

Different lowercase letters in the same column indicate significant differences between means, according to the least significant difference test (*p*<0.05). Values represent means±SD (*n*=3).

The A-ring and B-ring in SLs can be modified through methylation, hydroxylation, epoxidation, or ketolation, giving rise to the structural plasticity of SLs that often results in changes in their biological activity (Thuring et al., 1997c; Boyer et al., 2012; Al-Babili and Bouwmeester, 2015). In the case of 7BrGR24 and 7FGR24, halogenation of the A-ring at the C-7 increased the germination of *O. cumana* 3- and 5-fold, respectively, compared to (+)-GR24 (*p*<0.05). Hence, the 7FGR24 showed the highest promotive activity achieving germination of 62.0±9.1% at 1.0×10^−8^M and reaching 90.9±3.8% at 1.0×10^−6^M. Previous reports indicated that the incorporation of substitutions in the A-ring and varying sizes of the side groups modified the promotive activity of the (+)-GR24 molecule on the germination of parasitic seeds. For example, (+)-GR24 analogs monohydroxylated in the A-ring from C-8 to C-5 were reported less active than (+)-GR24 on *S. hermonthica*, with a stronger fall in activity, when the hydroxyl group was closer to the bioactiphore part of the molecule (Ueno et al., 2011). However, the introduction of a hydroxyl group on the A-ring enhanced the germination-stimulating activity on *O. minor*, where a hydroxyl group is preferable at C-9 instead of at C-5 (Kim et al., 2010). Furthermore, the 6-methyl substituent on (+)-GR24 resulted in higher percentages of germinated *O. crenata* seeds (Wigchert and Zwanenburg, 1999). Moreover, bulky side groups joined to the A-ring also reduced the activity of SL analogs more than small groups (Cohen et al., 2013). Accordingly, the germination-stimulating activity of SLs depended on both the position and size of the substituent on A-ring. Although, a few of reports declared the introduction of substituent such as iodine atom to the A-ring at the C-7 reduced the activity of SL analogs on *O. crenata* and *Pisum sativum* (Thuring et al., 1997c; Boyer et al., 2012). In our results, 7FGR24 showed higher activity. It might be due to the fact that both the A-ring halogenation at the 7-C position, which was far from the GR24 bioactiphore and the small size of the fluorine atom likely favored a high affinity of 7FGR24 to the active site of SLs receptors of *O. cumana*.

### YLG Assay

**Figure 4:**
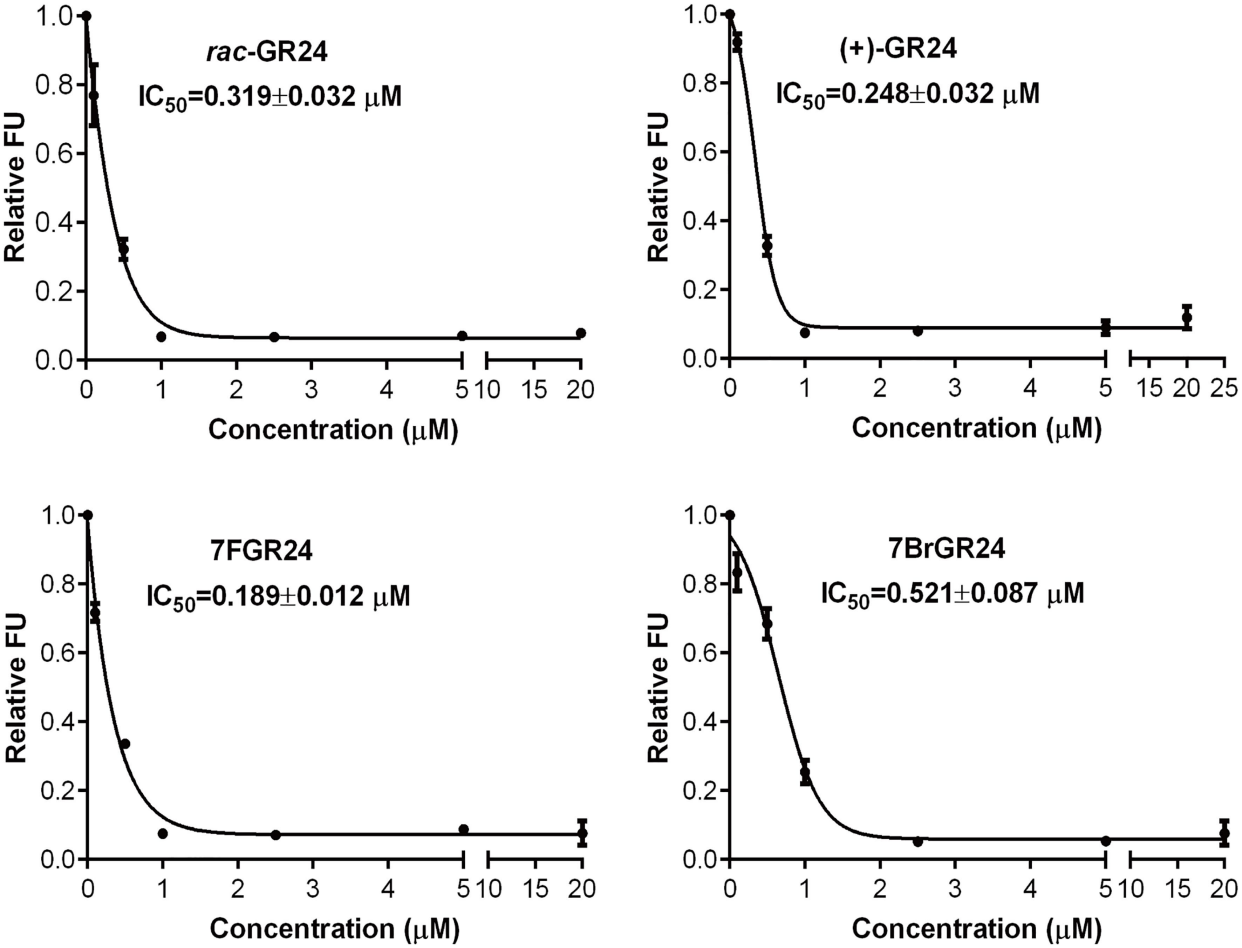
Relative fluorescence unit (FU) values were recorded for *rac*-GR24, (+)-GR24, and the halogenated (+)-GR24 analogs when tested at several concentrations in the yoshimulactone green (YLG) assay. IC_50_ values for these strigolactone (SL) analogs were calculated. Values represent means±SD (*n*=4).

(+)-GR24 and its halogenated analogs showed binding affinity to ShHTL7, an SL receptor found in the parasitic plant *S. hermonthica*, with a high affinity to SLs (Tsuchiya et al., 2015). Binding affinity was tested by an *in vitro* fluorescence-based competition assay involving YLG. The YLG was a small probe that emits fluorescence only after the hydrolysis, which was catalyzed by ShHTL7. A decrease in FUs showed the competition for receptor binding between a fixed YLG concentration and increasing concentrations of the SL analog. The halogenated GR24 analogs 7BrGR24 and 7FGR24 tested at concentrations between 2.5 and 20μM showed approximately 0.1 FU, which were similar to those recorded for (+)-GR24 and *rac*-GR24 (Figure 4). The FU of 7FGR24 were below 0.34 as in the case of (+)-GR24 and *rac*-GR24, even at a concentration range from 0.5 to 1.0μM. Moreover, 7FGR24 tested at 0.1μM was 0.72 FU, which was significantly lower than 0.81 and 0.92 FU recorded for *rac*-GR24 and (+)-GR24, respectively (*p*<0.05). Probit analysis based on FUs indicated that 7FGR24 was the strongest competitor tested (IC_50_=0.189±0.012μM), followed by (+)-GR24 (IC_50_=0.248±0.032μM), whereas *rac*-GR24 and 7BrGR24 had a lower affinity for ShHTL7 with IC_50_ values of 0.319±0.032 and 0.521±0.087μM, respectively. Consistent with this, the substituent at C-8 also showed higher affinity for ShHTL7 (Tsuchiya et al., 2015). Although, the 7BrGR24 posed lower affinity, as compared to (+)-GR24, which was inconsistent with seed germination activity. The discrepancy could be due to the fact that the that ShHTL7 protein was derived from a *Striga* ssp. not an *Orobanche* ssp., both of which could respond differently to 7BrGR24.

### Molecular Docking Assays

**Figure 5:**
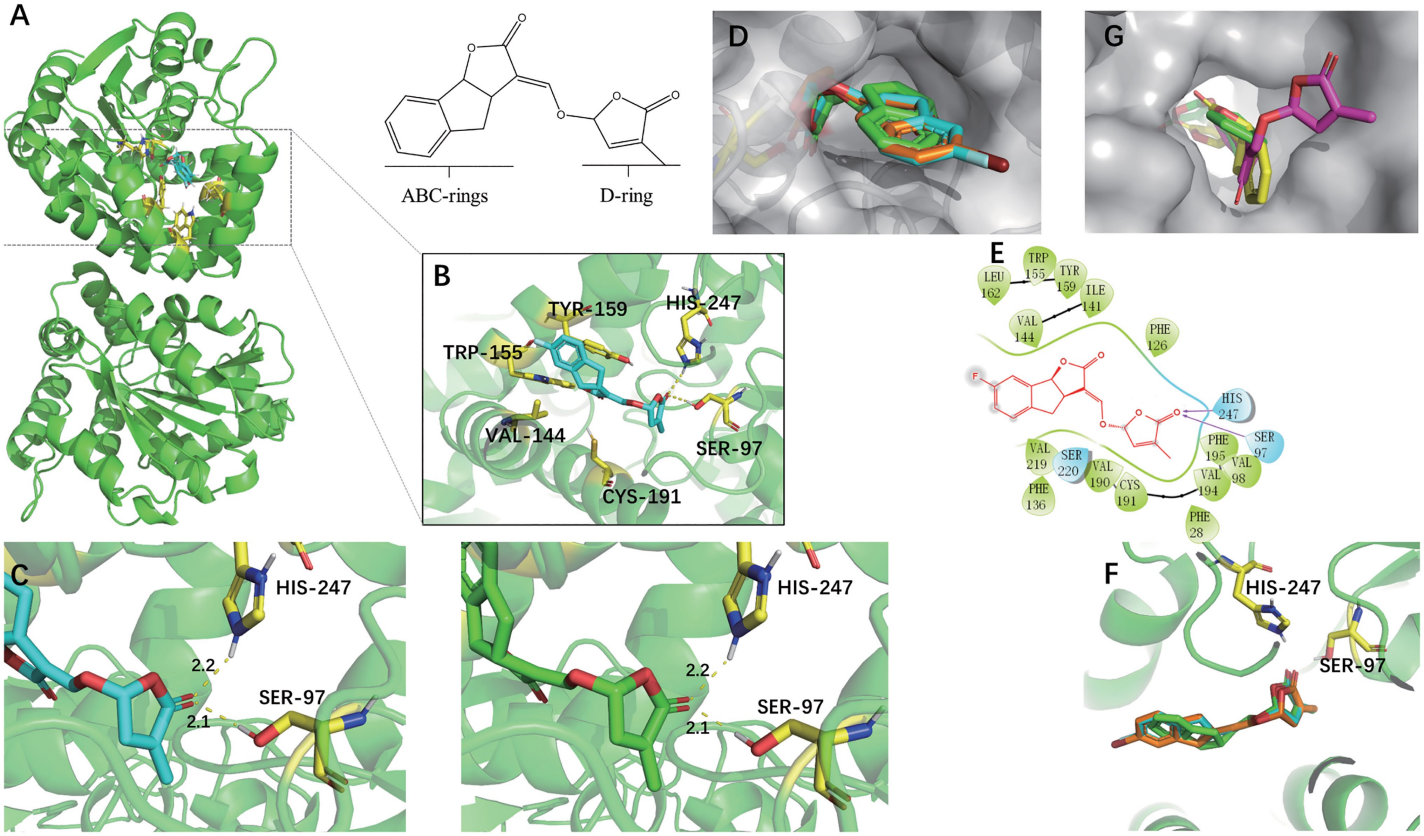
Docking modes of (−)-epi-GR24 and the halogenated (+)-GR24 analogs with OsD14. **(A)** Pocket location of OsD14 when 7FGR24 combined on it. **(B)** Spatial position and interaction force between 7FGR24 and important amino acid residues in the active pocket. **(C)** The distance of the polar connection between the 7FGR24/(+)-GR24 and Ser 97 and His 247. **(D)** The halogenated (+)-GR24 analogs in the active pocket (the yellow sticks in the pocket are SER97). **(E)** 2D view of 7FGR24 in the catalytic site of OsD14. **(F)** Comparison of binding postures of 7FGR24, 7BrGR24, and (+)-GR24. **(G)** Two ways of connecting (−)-epi-GR24 to active pocket. The (−)-epi-GR24 is shown as yellow and purple sticks, 7BrGR24 as orange sticks, 7FGR24 as blue sticks, and the original ligand of OsD14 – (+)-GR24 as green sticks.

Rice DWARF14 (OsD14) was selected for docking studies in order to understand how the SL analogs interacted with the SL receptor. SLs receptors were AtD14 paralogs forming part of the α, β-fold hydrolases family, which not only binded to the SL molecules but also cleaved them into their ABC-ring and the D-ring parts (Hamiaux et al., 2012). They were structurally similar and had a conserved catalytic pocket consisting of a triad of serine, histidine, and aspartate (Yao et al., 2016). The docking analyses indicated that (+)-GR24 and the halogenated (+)-GR24 analogs could smoothly enter the binding pocket of the OsD14 protein (Figure 5). Their D-rings acquire the same orientation predicted for (+)-GR24 during its interaction with the receptor (Trott and Olson, 2010; Figure 5D). As shown in Figure 5B, the carbonyl oxygen in the D-ring of 7FGR24 formed hydrogen bonding forces with Ser97 and Hip247, which were part of the OsD14 catalytic triad. The polar connection between the hydroxyl hydrogen atom in Ser97 and the carbonyl oxygen in the D-ring of SLs was a key step required for the successful hydrolysis of SLs (Kagiyama et al., 2013). Further predictions obtained for these two hydrogen bonds in the enzyme-catalyzed reactions revealed that their distances and positions were similar to those expected for the ligation of (+)-GR24 (Figure 5C). This should be responsible for the high biological activity observed in 7FGR24. It was worth noting that, the fluorine atom could modify physicochemical properties of the GR24 analog, such as pKa and lipophilicity, improving its permeability through cell membranes (Purser et al., 2008). Moreover, as shown in Figure 5F, the posture of the D ring in 7FGR24 was obviously more similar to the original ligand of the crystal structure-GR24, which meant that 7FGR24 could be more conducive to hydrolysis, compared to 7BrGR24. Furthermore, these different postures could be related to the distinct atomic radii and electronegativities of observed between atoms F and Br.

In addition, we also conducted docking experiments on (-)-epi-GR24, which was inactive on seed germination. The (-)-epi-GR24 had two main binding poses differing from each other in the location of the D-ring. One pose showed the D-ring into the active site, while the other revealed the ABC-ring positioned into the active pocket with its D-ring in an outer location (Figure 5G). In both cases, D-ring orientation was different from the expected during (+)-GR24-OsD14 interaction. Binding energies calculated for the poses of (-)-epi-GR24 were near to the binding energy predicted for (+)-GR24. Hence, both bindings of (-)-epi-GR24 were possible, although, the D-ring would be not properly oriented for the hydrolytic cleavage at the enol–ether bond catalyzed by OsD14. In addition, the docking analyses for (+)-GR24 and 7BrGR24 were similar to those obtained for 7FGR24.

## Conclusion

Two halogenated (+)-GR24 analogs (7BrGR24 and 7FGR24) were synthesized through a relatively short number of synthetic steps and their promotive effect were tested on seed germination of *O. cumana*. Both stimulated its germination and showed a binding affinity for the SL receptor protein ShHTL7. However, 7FGR24 was the strongest germination promoter tested and had the highest binding affinity to ShHTL7. Molecular docking assays supported structural features of 7FGR24, which explained the higher activity compared to that of *rac*-GR24 and (+)-GR24. Our results indicate that 7FGR24 is a promising agent for the control of parasitic weeds.

## Data Availability Statement

The original contributions presented in the study are included in the article/Supplementary Material, further inquiries can be directed to the corresponding authors.

## Author Contributions

YY, LH, and SY conceived and designed the experiments. YK, LS, and XW designed and synthesized the analogs. YC, YK, LS, and XW assisted and performed the experiments. YC, YK, LS, XW, HF, SY, DS, LH, and YY wrote the manuscript and respective parts. YY and SY supervised the study. All authors contributed to the article and approved the submitted version.

## Funding

This research was funded by the Ability Establishment of Sustainable Use for Valuable Chinese Medicine Resources (2060302), the National Science & Technology Fundamental Resources Investigation Program of China (2018FY100800), the Fundamental Research Funds for the Central Public Welfare Research Institutes (ZZ10-008), National Natural Science Foundation of China (81891013, 81891010, and 21702187), and Scientific and Technological Innovation Project of China Academy of Chinese Medical Sciences (CI2021A041).

## Conflict of Interest

The authors declare that the research was conducted in the absence of any commercial or financial relationships that could be construed as a potential conflict of interest.

## Publisher’s Note

All claims expressed in this article are solely those of the authors and do not necessarily represent those of their affiliated organizations, or those of the publisher, the editors and the reviewers. Any product that may be evaluated in this article, or claim that may be made by its manufacturer, is not guaranteed or endorsed by the publisher.

## Supplementary Material

Supplementary Figure S1 ^1^H NMR of 7BrGR24.

Supplementary Figure S2 ^13^C NMR of 7BrGR24.

Supplementary Figure S3 ^1^H NMR of 7FGR24.

Supplementary Figure S4 ^13^C NMR of 7FGR24.
