# Supplementary figures and images for: Synthesis and Evaluation of New Halogenated GR24 Analogs as Germination Promotors for Orobanche cumana
Source: Front Plant Sci. 2021 Sep 17;12:725949. doi: 10.3389/fpls.2021.725949 (PMC8484532; doi:10.3389/fpls.2021.725949)

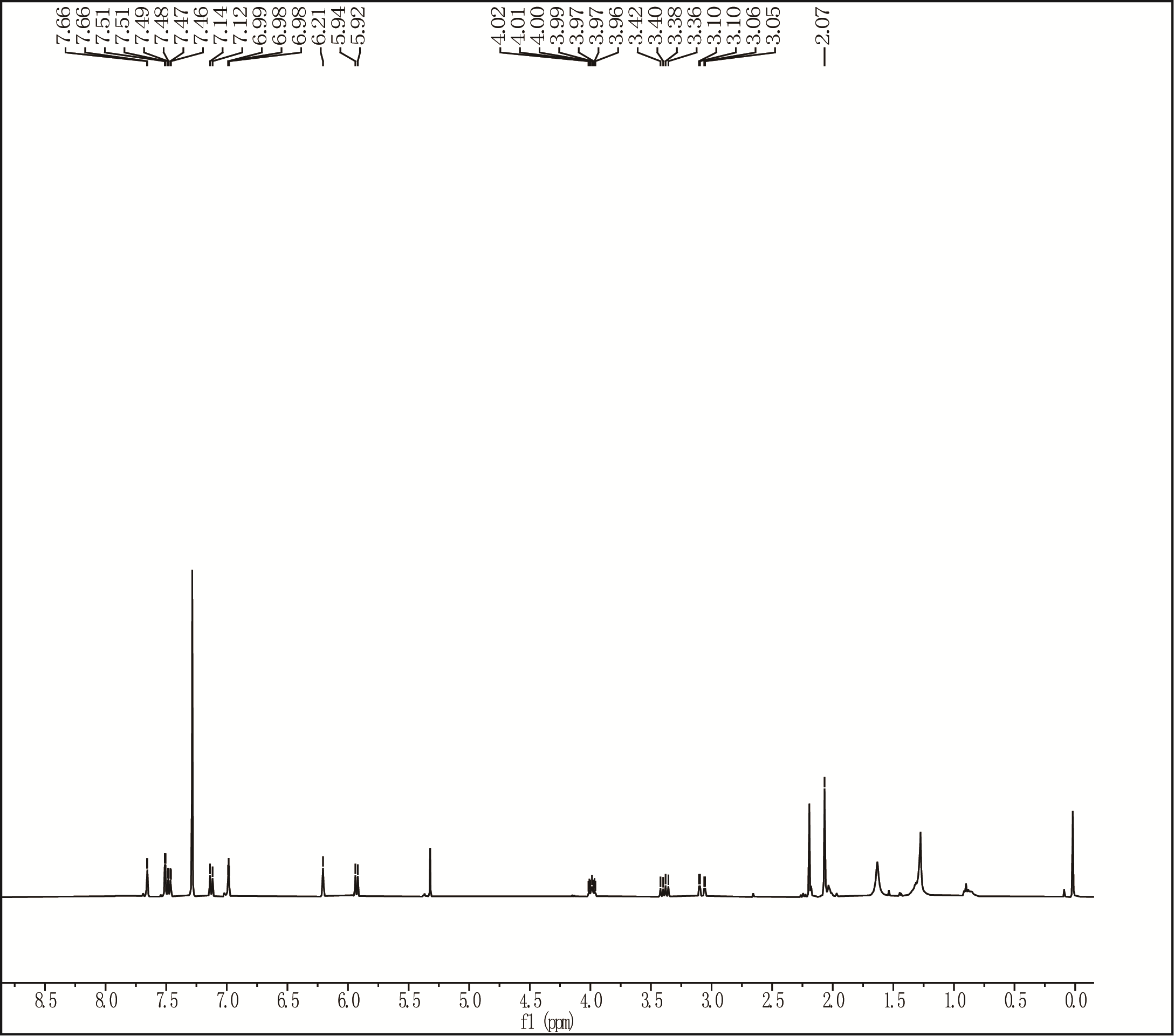

Supplement: Supplementary Figure S1 — 1H NMR of 7BrGR24. [file Image_1.TIF]

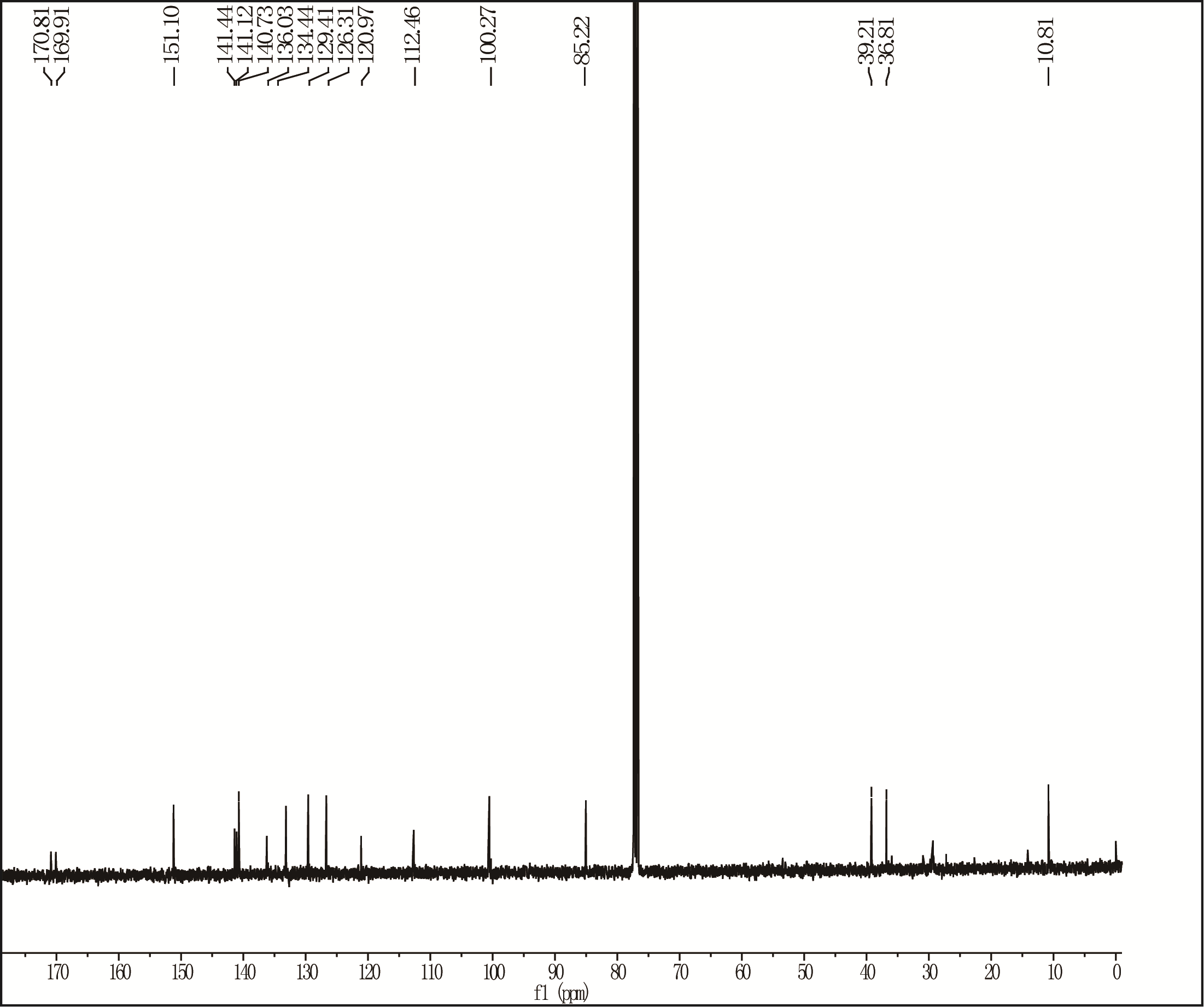

Supplement: Supplementary Figure S2 — 13C NMR of 7BrGR24. [file Image_2.TIF]

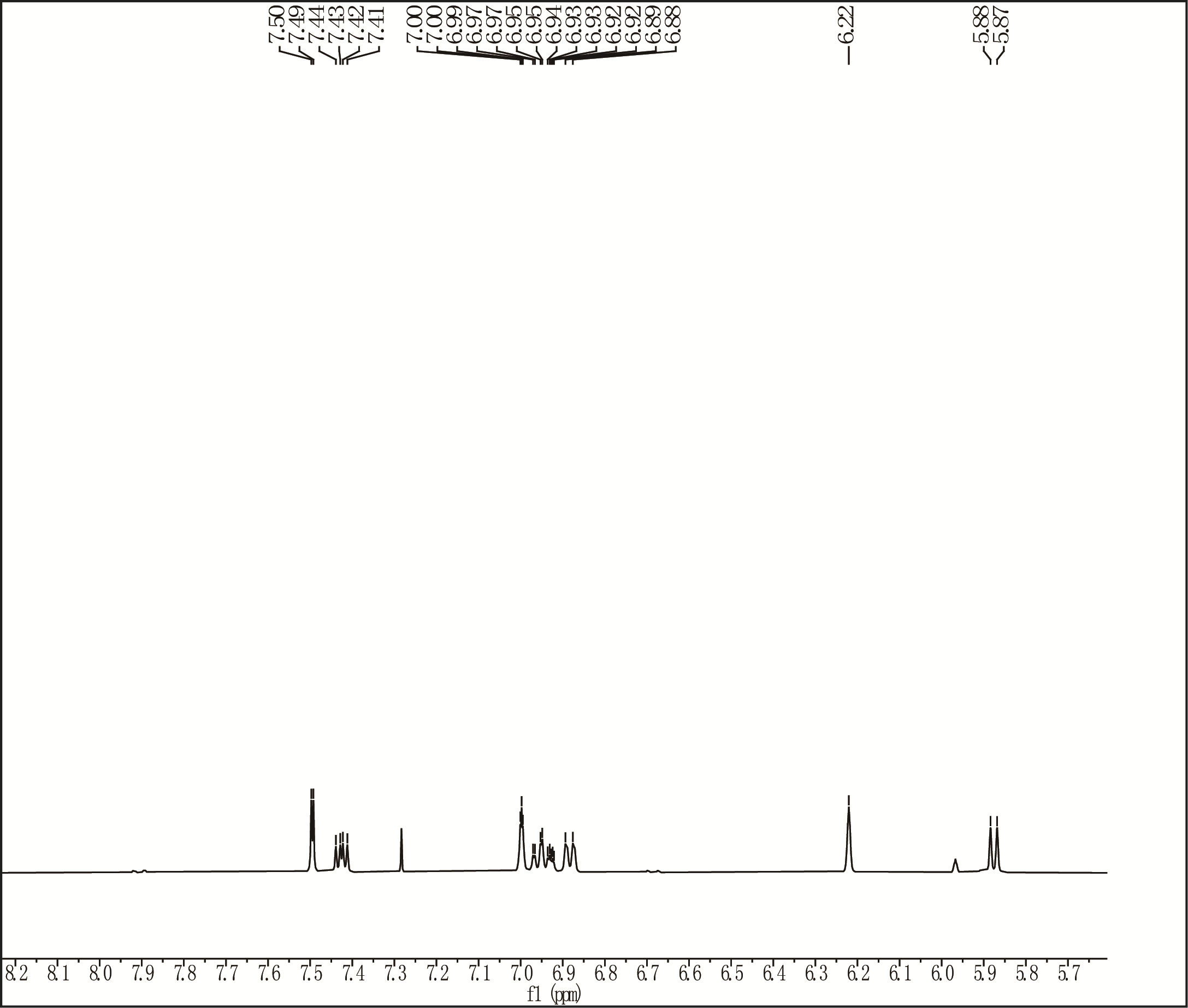

Supplement: Supplementary Figure S3 — 1H NMR of 7FGR24. [file Image_3.TIF]

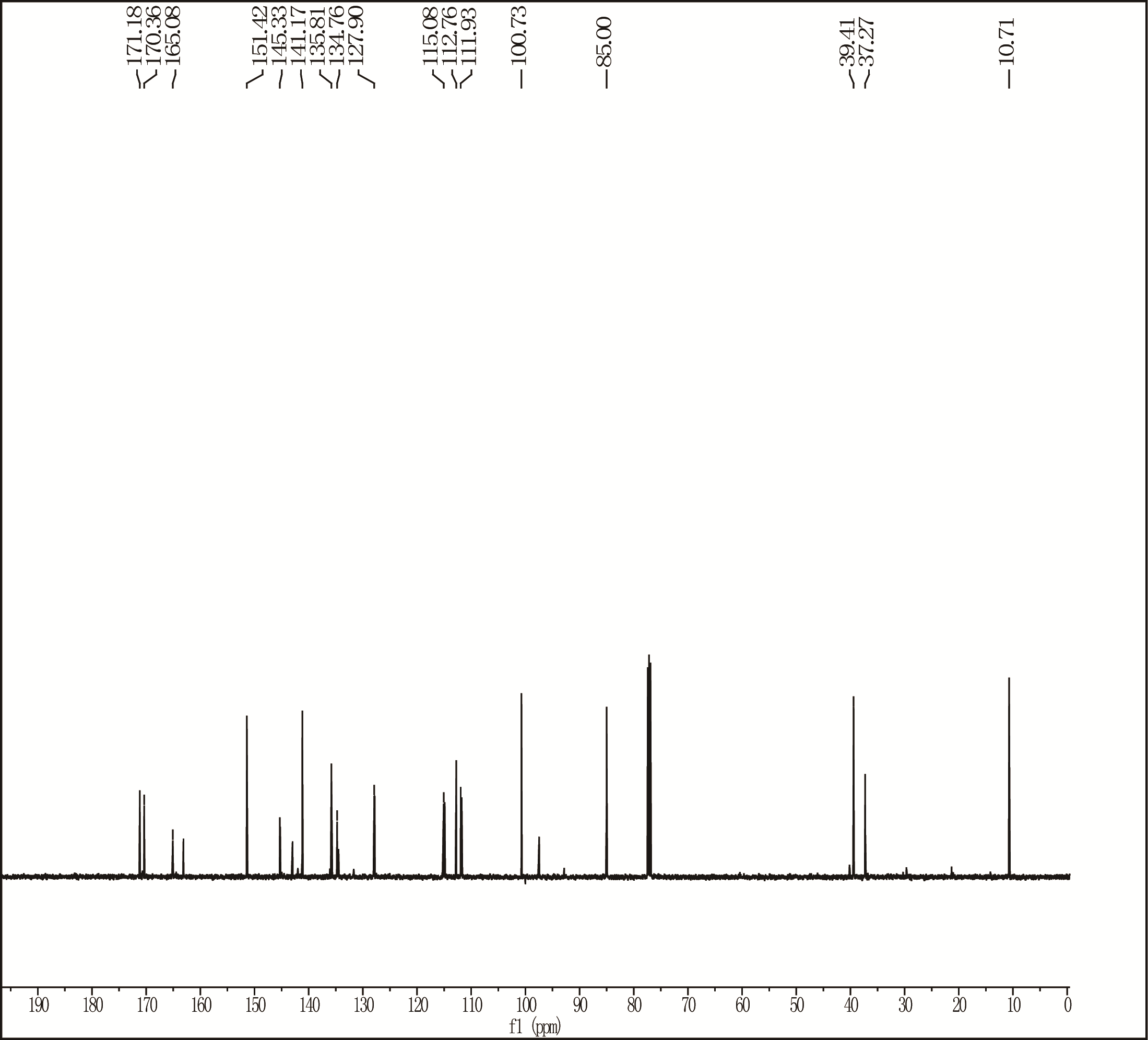

Supplement: Supplementary Figure S4 — 13C NMR of 7FGR24. [file Image_4.TIF]
